# The effects of oral iron supplementation on cognition in older children and adults: a systematic review and meta-analysis

**DOI:** 10.1186/1475-2891-9-4

**Published:** 2010-01-25

**Authors:** Martin Falkingham, Asmaa Abdelhamid, Peter Curtis, Susan Fairweather-Tait, Louise Dye, Lee Hooper

**Affiliations:** 1 Diet and Health Group, School of Medicine, Health Policy and Practice, University of East Anglia, UK; 2 Human Appetite Research Unit, Institute of Psychological Sciences, University of Leeds, UK

## Abstract

**Background:**

In observational studies anaemia and iron deficiency are associated with cognitive deficits, suggesting that iron supplementation may improve cognitive function. However, due to the potential for confounding by socio-economic status in observational studies, this needs to be verified in data from randomised controlled trials (RCTs).

**Aim:**

To assess whether iron supplementation improved cognitive domains: concentration, intelligence, memory, psychomotor skills and scholastic achievement.

**Methodology:**

Searches included MEDLINE, EMBASE, PsychINFO, Cochrane CENTRAL and bibliographies (to November 2008). Inclusion, data extraction and validity assessment were duplicated, and the meta-analysis used the standardised mean difference (SMD). Subgrouping, sensitivity analysis, assessment of publication bias and heterogeneity were employed.

**Results:**

Fourteen RCTs of children aged 6+, adolescents and women were included; no RCTs in men or older people were found. Iron supplementation improved attention and concentration irrespective of baseline iron status (SMD 0.59, 95% CI 0.29 to 0.90) without heterogeneity. In anaemic groups supplementation improved intelligence quotient (IQ) by 2.5 points (95% CI 1.24 to 3.76), but had no effect on non-anaemic participants, or on memory, psychomotor skills or scholastic achievement. However, the funnel plot suggested modest publication bias. The limited number of included studies were generally small, short and methodologically weak.

**Conclusions:**

There was some evidence that iron supplementation improved attention, concentration and IQ, but this requires confirmation with well-powered, blinded, independently funded RCTs of at least one year's duration in different age groups including children, adolescents, adults and older people, and across all levels of baseline iron status.

## Introduction

Anaemia, defined as 'a reduction in the quantity of the oxygen-carrying pigment haemoglobin in the blood'[1], is a major global public health problem. It is estimated that 25% of the world's population have anaemia, and approximately 50% of cases are due to iron deficiency [2] where the anaemia is caused by an inadequate supply of iron to form haemoglobin (Hb). Lower concentrations of Hb result in a number of symptoms such as weakness and general fatigue, and adverse effects on the immune system [3]. In more severe cases a need to increase cardiac output leads to dyspnoea (shortness of breath), palpitations and heart failure, and in pregnancy an increased risk of pre-term delivery and low birth weight [4].

Many factors may contribute to the risk of developing iron deficiency anaemia (IDA), including low iron intake and poor absorption of iron (from diets high in iron chelators such as phenolic compounds and phytate, or low in ascorbic acid and meat/fish), and high iron demand (during menstruation, pregnancy and growth spurts). These result in a higher risk of IDA at 6-12 months of age, during adolescence (especially in girls at the onset of menstruation), women of child-bearing age (especially during pregnancy), and older people (when diets may be less nutritious and malabsorption increases) [2]. There is also a higher risk of anaemia in the presence of chronic inflammatory states, which are common in the elderly, mediated by raised hepcidin expression. Additional risk factors include heavy menstrual blood loss, parasitic infections, acute and chronic infections, other micronutrient deficiencies, and haemoglobinopathies [2].

Cognition is defined as 'The mental processes by which knowledge is acquired. These include perception, reasoning, acts of creativity, problem solving, and possibly intuition.'[1] Cognition is important for quality of life, such that impaired cognitive function is correlated with poorer quality of life e.g. in stroke patients [5] and poorer life prospects.

The effect of iron supplementation on a range of health outcomes in infants and young children has been well explored. It is estimated that 47% of pre-school children worldwide have anaemia, the highest prevalence of any population group [2]. Longitudinal studies show that iron deficiency in infancy is related to poorer cognition in childhood [6]. One systematic review that included seven RCTs on the effects of supplementary iron in young children with anaemia or iron deficiency found no evidence of an effect of iron supplementation on psychomotor development [7], while another including seventeen RCTs in children of any age and with any initial iron status, found that iron supplementation was not associated with improved mental development scores in children under 5 years [8], or with improved physical growth [9]. A more recent systematic review addressed a range of health risks and benefits of iron supplementation in infants and children aged under 5 years [10], finding that supplementation led to improvements in cognition and motor development in anaemic and iron-deficient children, but was associated with increased risk of death in areas with endemic malaria. As animal studies have shown that in the developing brain iron deficiency is associated with hypomyelination of neurones [11], effects on the dopaminergic system and a deficiency of enzymes involved in the development of parts of the brain important for cognitive functions such as memory (e.g. the hippocampus) [12], deficiency and supplementation may have different effects on infants and young children than in other population groups. For this reason, and because there are already several reviews covering this group, we have excluded studies on infants and young children from this review.

Older children and adolescents are less at risk of anaemia than pre-school children, but global statistics indicate that approximately 25% of older children have anaemia, as do, 30% of non-pregnant women and 42% pregnant women, and 17% of elderly people (rising to 40-50% of those admitted to hospital or living in nursing homes), demonstrating that it is a very large and important health problem [2,13]. While observational evidence suggests a strong link between iron deficiency or anaemia and cognitive deficit, the evidence of a causal link through intervention studies is limited. In order to maximise the power of the conducted RCTs to address the effect of iron supplementation, we conducted a systematic review and meta-analysis of the literature. This study aims to pool data from all available high quality RCTs to ascertain whether there is a beneficial effect of iron supplementation on cognition in humans aged 6 years and above, whether this differs according to baseline iron status, and whether it is different in various age groups. The systematic review is presented in a form consistent with the PRISMA Statement (see Additional File 1), no protocol for this review has been published or registered[14]

## Materials and methods

### Search methods

Structured electronic searches were carried out from inception to November 2008 on MEDLINE, EMBASE (both on Ovid), PsychINFO and Cochrane CENTRAL. The search included text and indexing terms, truncation and Boolean operators in the format ' [cognition text and indexing terms] and [iron text and indexing terms] and [RCT filter]'. The full MEDLINE search is shown in Additional File 2. The search was not limited by language. At least two reviewers each independently scanned identified titles and abstracts, ordering papers that either reviewer felt might fulfil the inclusion criteria. The reference lists of included studies and relevant reviews were also checked for relevant studies. Several authors were contacted to query inclusion criteria, and one study [15] was included on this basis.

### Inclusion criteria, data extraction and validity assessment

The inclusion criteria were that participants were human and aged at least 6 years, participants had to be randomised to an iron supplementation (as a fortified food or a supplement) vs. a control (placebo or no intervention) arm, duration of intervention was at least 4 weeks, the additive effect of iron had to be clear (so multiple nutrient supplements compared with no supplementation was not acceptable), and some objective measure of cognitive performance had to be assessed.

At least two reviewers independently assessed each full text study for inclusion, and disagreements were settled through group discussion. Papers were grouped into individual studies, and then data extraction and assessment of validity of studies were carried out independently at least in duplicate (sometimes triplicate) onto a standardised data extraction form. The completed forms were discussed within the group, and disagreements settled with reference to a third reviewer or the wider team. Data extracted included methodological details, participant characteristics and flow, intervention and control details (including type and dose of iron, as well as similarity to the control), outcome data; including primary outcomes (measurements of cognition), secondary outcomes (e.g. adverse effects or side effects of intervention, and changes in serum Hb and serum ferritin (SF)), and issues to check with study authors.

Primary outcomes were characterised as belonging primarily to one of the following cognitive domains: intelligence, memory, concentration or psychomotor skills, or to scholastic achievement which may reflect a mixture of these skills, and is the most ecologically valid measure of performance. Where studies provided several different tests that fell within a single domain the outcome used in analysis was that which was shared with other published studies. For scholastic achievement outcomes testing mathematical skills were prioritised to enhance the objectivity of measurement. For continuous primary and secondary review outcomes mean change in the outcome from baseline to latest duration, standard deviation of that change and the number of participants were recorded for the intervention group and control group. Where change data were not provided, absolute measurements from the end of the intervention period were recorded in their place. Where variances were provided as standard errors they were converted to standard deviations, and where tests showed better cognition with a lower score the signs of the means were reversed. Where data were provided in subgroups (for example for those anaemic or not anaemic at baseline), these data were extracted and used in preference to grouped data. In studies where two different doses or frequencies of iron supplementation were used with only one control group then data from the two intervention arms were combined using the methods recommended in the Cochrane Handbook [16].

Assessment of validity was included in the main data extraction form and included whether randomisation was described, allocation concealment, masking of the participants, researcher(s) and outcome assessor(s), change in iron status (described positively where there were statistically significant differences in Hb or SF between iron and placebo groups at the end of the study OR, if there was no information provided on this, the intervention duration was at least 12 weeks), inclusion of all those randomised in the outcomes, and potential funding bias.

Authors of studies which did not contain sufficient data to be included in data analysis were contacted via e-mail and by letter and asked to provide raw outcome data from their study.

### Analysis

Data on the characteristics and validity of the included studies were tabulated (see Tables 1 and 2). The computer software 'Review Manager 5' was used to meta-analyse data within the cognitive domains of intelligence, memory, concentration, scholastic achievement and psychomotor skills. Subgrouping was by baseline iron status (as defined by the World Health Organisation) and by age group [17]. Anaemia was defined as Hb levels <11.5 g/dl in children aged 6-12, <12 g/dl children aged 13-14 and non-pregnant adult women, <13 g/dl in adult men and <11 g/dl in pregnant women. Iron deficiency was defined as not having anaemia, but having SF <15 μg/L, the remainder were considered to be iron replete at baseline. Where SF was not reported and participants were not anaemic they were categorised as 'iron deficient and/or replete'. Study populations were described as children (aged 6-18), pre-menopausal women, post-menopausal women, or men.

**Table 1:** Characteristics of included studies.

Study Name	n	Population	Dose & type of oral iron	Baseline Iron Status	Study durat^n^, Drop-outs	Cognitive tests*
**Beard 2005 **[19,45]	Iron 34 Pl 30	S Africa-mothers, 18-30 yrs **Pre-menopausal women**	125 mg/d as pills	**Anaemic **- Hb 9-11.5 g/dl, SF 10-12 μg/L	29 wks, Iron 2 Pl 4	**Raven's CPM (IQ), Wechsler's DS (M, Ps)**

**Bruner 1996 **[30,46]	Iron 40 Pl 41	USA-adolescents, school, mean age 16.2 and 15.7 **Children 6-18**	260 mg/d EFe as capsules	**Iron Defic **- Hb>11.5 g/dl (Af American), Hb>12 g/dl (white), SF<12 μg/L for all	8 wks, Iron 1 Pl 2	Visual Search and Attention (AC), Hopkin's Verbal Learning Test (M), DS Modalities (Ps), Attention.

**Elwood 1970 **[20]	Iron 26 Pl 21	UK - women >20 yrs **Pre-menopausal women**	150 mg/d as tablets	**Anaemic **- HB<10.5 g/dl	8 wks, Iron 0 Pl 0	**Mazes test (AC), Serial Sevens (M), Peg board (Ps), **E test, card sorter.

**Gopaldas 1985 **[21,47-49]	Iron 32 Pl 16	India-School boys-recipients of free noon meal, 8-15 yrs **Children 6-18**	30 or 40 mg/d as tablets	**Anaemic **- Hb<10.5 g/dl, **Iron Defic and/or Repl- **rest	8.5 wks, Iron 0 Pl 0	**Mazes test (AC), Visual Memory Test (M), Wechsler's Digit span (Ps), **Clerical task

**Groner 1986 **[22]	Iron 19 Pl 19	USA-pregnant women, 14-24 yrs **Pre-menopausal women**	60 mg/d EFe as capsules	**Iron repl **- mean Hb>12 g/dl, SF 40-60 μg/L	4 wks, Iron 3 Pl 10	**Vocab (IQ), DS (M, Ps), Arithmetic (SA), **Consonant trigram, Rey AVL, Digit Span

**Kashyup 1987 **[23,49]	Iron 83 Pl 83	India-School girls, under privileged, 8-15 yrs **Children 6-18**	60 mg/d EFe as tablets	**Anaemic **- Hb<10.5 g/dl, **Iron Defic and/or Repl**-rest	17.2 wks, Iron 18 Pl 18	**Mazes test (AC), Visual Memory (M), Wechsler's Digit span (Ps), **Clerical

**Kordas 2005 **[32,50,51]	Iron 303 Pl 299	Mexico-school children, mean age 7.0 **Children 6-18**	30 mg/d as tablets	**Iron Defic and/or Repl **- Hb>9 g/dl	21 wks, Iron 38 Pl 49	Distractibility (AC), Peabody PV (IQ), Vis Memory (M), Maths (SA), Sternberg**

**Lambert *et al*., 2002 **[27]	Iron 57 Pl 59	New Zealand-female high school students, 12.5-17.9 yrs **Children 6-18**	105 mg/d EFe as tablets	**Iron defic **- Hb>12 g/dl, SF<12 μg/L	8 wks, 5 across whole study	Visual Search and Attention (AC), Hopkin's Verbal Learning (M), Stroop task, Reading span

**Lynn & Harland 1998 **[15]	Iron 208 Pl 205	England - teenagers at 7 comprehensive schools, 12-16 yrs **Children 6-18**	17 mg/d EFe as tablets	**Iron Defic **- any Hb, SF<12 μg/L ??and??? **Iron Repl **- the rest	16 wks, Unclear, ~200 over study?	**Raven's CPM (IQ)**

**Murray-Kolb *et al *2007 **[31]	Iron 76 Pl 76	USA-Women, aged 18-35 yrs **Pre-menopausal women**	60 mg/d EFe as pills	**Anaemic **- Hb 10.5-11.9 g/dl plus 2 aFeSIs, **Iron Defic **- Hb = 12 g/dl plus 2 aFeSIs, **Iron Repl **- Hb = 12 g/dl without 2 aFeSIs	16 wks, Iron 20 Pl 19	Cog Abilities-attention (AC), Cog Abilities - memory (M), Cog Abilities - learning (SA), Shipley Inst Scale (IQ)

**Pollitt 1989 **[24]	Iron 678 Pl 678	Thailand-school children, 9-12 yrs **Children 6-18**	4 mg/kg/d EFe as tablets	**Anaemic **Hb<12 g/dl and SF<10 μg/L or TS<16%, **Iron Defic **Hb>12 g/dl and SF<10 μg/L or TS<16%, **Iron Repl **- rest	16 wks, Not reported	**Raven's CPM (IQ), Mathematics (SA), **Thai language

**Soemantri 1985 **[29,52]	Iron 59 Pl 60	Indonesia-primary school children, mean age 10.7 to 11.1 yrs **Children 6-18**	2 mg/kg/d EFe as tablets	**Anaemic **- Hb<11 g/dl, TS<15%, **Iron Repl **- Hb>12 g/dl, TS>20%	13 wks, Iron 0 Pl 0	Raven's CPM (IQ), Bourden-wisconsin concentration, Maths, Language, Biology, social science

**Soemantri 1989 **[25]	Iron 71 Pl 59	Indonesia-primary school children, mean age 10.4 **Children 6-18**	2 mg/kg/d EFe tablets	**Anaemic **- Hb<11 g/dl, TS<12%, **Iron Repl **- Hb>12 g/dl. TS <20%	13 wks, Iron 0 Pl 0	**Raven's CPM (IQ), Maths (SA), **Language, Biology, social science

**Sungthong 2004 **[26,53]	Iron/d 112 Iron/wk 102 Pl 99	Thailand-School children, mean age 9.6 to 9.7 **Children 6-18**	60 mg/d or/wk EFe, tablets	**Iron Repl **- Hb>8 g/dl, SF>20 μg/L	16 wks, Iron/d 1 Iron/wk 4 Pl 1	**Test of Non-Verbal Intelligence (IQ), Maths (SA), **Thai Language

*tests in bold have been used in the analysis

** Plus Figure match & design, Stimulus discrimination, Cognitive abilities, Visual search, Sequencing

Hb: haemoglobin, TS: transferrin saturation, SF: serum ferritin, EFe: elemental iron, aFeSI: abnormal iron status indicators, Pl: placebo, AC: Attention and concentration, IQ: Intelligence quotient, M: memory, Ps: psychomotor, SA: Scholastic Achievement, Raven's CPM: Raven's Colour Progressive Matrices, Peabody PV: Peabody Picture Vocabulary, Rey AVL: Rey Auditory Verbal Learning, Digit Symbol: DS, Repl: repletion, Defic: deficient

**Table 2:** Validity characteristics of included studies

	Random-isation Described/Allocation Concealment	Researcher/Outcome Assessor/Participants Masked to intervention	Change in iron status OR 12+ wks?*	All those randomised included in outcomes?/Reason for dropouts reported?	Potential for funding bias**	Study data useable in meta-analysis?
**Beard 2005**	Partially/Unclear	Unclear/Unclear/Yes	**Yes **(SF, 29 wks)	No/Partially	Moderate	Yes

**Bruner 1996**	Yes/Unclear	Yes/Unclear/Yes	**Yes **(Hb & SF)	No/Yes	High	No (no raw data, only regression)

**Elwood 1970**	No/Unclear	Unclear/Yes/Yes	**Unclear **(p-values not presented)	No/Partially	High	Yes

**Gopaldas 1985**	Partially/Unclear	No/No/Yes	**Anaemic & Iron repl: Yes **(Hb)	Yes/Yes (no drop-outs)	High	Yes

**Groner 1986**	No/Unclear	Yes/Yes/Yes	**Yes **(Hb)	No/Yes	Moderate	Yes

**Kashyap 1987**	Partially/Unclear	Unclear/Unclear/Yes	**Anaemic & Iron repl: Yes **(Hb, 17 wks)	No/Partially	Moderate	Yes

**Kordas 2005**	Yes/Unclear	Yes/Yes/Yes	**Yes **(21 wks)	No/Partially	Moderate	No (only z-scores)

**Lambert 2002**	No/Unclear	Yes/Unclear/Yes	**Yes **(SF, not Hb)	No/No	Moderate	No (no variance data)

**Lynn & Harland 1998**	Yes/Unclear	Yes/Yes/Yes	**Iron Defic & Iron repl: Yes **(16 wks)	Unclear/No	High	Yes

**Murray-Kolb 2007**	Yes/Unclear	No/Yes/Yes	**Anaemic & Iron repl: Yes **(SF, not Hb, 16 wks),**Iron defic: No **(not SF or Hb)	No/Partially	Moderate	No (reported only as z-scores)

**Pollitt 1989**	No/Unclear	Unclear/Unclear/Yes	**Anaemic, Iron defic & Iron repl: Yes **(Hb, SF, 16 wks)	No/No	Moderate	Yes

**Soemantri 1985**	No/Unclear	Unclear/Unclear/Unclear	**Anaemic: Yes **(Hb, 13 wks) **Iron repl: No **(Hb)	Unclear/No	Moderate	No (no variance or SD data)

**Soemantri 1989**	No/Unclear	Unclear/Unclear/Yes	**Anaemic & Iron repl: Yes **(Hb, 13 wks)	No/No	Moderate	Yes

**Sungthong 2004**	Yes/Yes	Yes/Yes/Yes	**Yes **(SF & Hb, 16 wks)	No/Yes	Low	Yes

* Iron status was said to have changed if there were statistically significant differences in Hb or SF between iron and placebo groups at the end of the study or, where no data were provided, if the intervention period was at least 12 weeks long.

** Potential for funding bias was assessed as follows: low (when funders were clearly not industry-related, no authors worked for industry and there was no suggestion that products were donated by industry); high (when non-industry funding was reported, or when funding was not mentioned); moderate in all other studies.

Meta-analysis used the inverse variance method. Because of the nature of the different cognitive test scoring systems, which used very different scales, standardised mean differences (SMD) were used in random effects meta-analysis. This allowed assessment of whether statistically significant effects were found in the pooled data, but did not provide outcome measures meaningful on any particular scale. Sensitivity analysis was employed to check the results of the meta-analyses, removing studies where it was not clear that iron status had altered during the study. The presence (or not) of publication bias was assessed using a funnel plot and studies that assessed outcomes that could not be included in the meta-analyses were discussed alongside the meta-analysis results. The importance of differences between studies, heterogeneity, was assessed using the I^2 ^statistic [18].

## Results

### Review process

**Figure 1:**
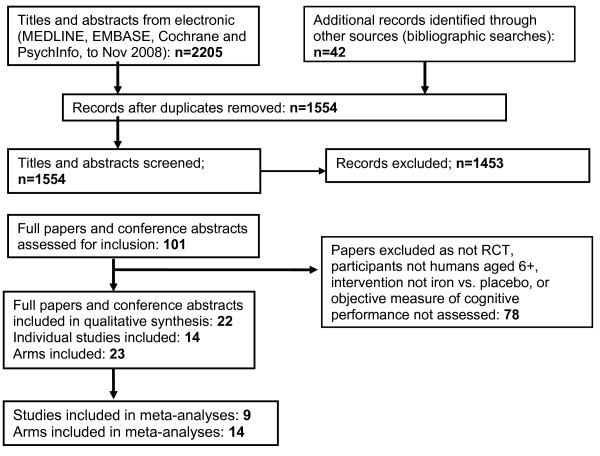
**PRISMA study flow diagram for the systematic review **[14].

From the 1554 titles and abstracts identified as separate papers (following de-duplication of the original 2247 records) via electronic and reference searching, 14 were included in the review, see Figure 1. Of these, 9 studies provided data which could be used in analysis [15,19-26] the others provided data as z-scores or without necessary data on variance. None of the studies identified required translation. We contacted authors of 9 of the 14 studies to request additional information, and all useful data received was included in the analysis.

### Study characteristics

The characteristics of the included studies are shown in Table 1 (including data from all relevant located publications of each study and any author information provided). Seven of the studies were carried out in developing countries (2 in Thailand, 2 in Indonesia, 1 in Mexico, 2 India) and 7 in developed countries (3 in the USA, 2 in the UK, 1 in South Africa and 1 in New Zealand). Most studies were carried out on children and/or adolescents, but studies also included pregnant women, mothers with young infants and anaemic non-pregnant women. No studies included men, post-menopausal women or the elderly, and no studies gave nutrients additional to iron in the intervention and placebo tablets. Studies ran from 4 to 29 weeks, so were of variable and relatively short duration.

The three forms of iron used were ferrous sulphate, ferrous carbonate and ferrous fumarate (one study did not mention the type of iron used [27]). All included studies used an oral iron supplement in the form of 'pills', 'capsules' or 'tablets', none gave supplemented foods.

A plethora of objective tests were used, measuring the specified domains of cognition (actual tests used in each study are detailed in Table 1). Tests were administrated by trained field workers, researchers or psychologists in three studies, a group of researchers and teachers or trained testers and school staff in two studies, by the school in one study and self-administered in one study (with no details reported in the remainder). In three studies the tests were administered individually while the remainder did not state group or individual administration. One study reported that the tests were completed with paper and pencil, one had computerised tests, one had verbal and computer testing, and one a mixture of paper and computerised formats.

### Validity of studies

Study validity is reported in Table 2 (including data from all available publications and contact with authors). The process of randomisation was described in 5 of 14 studies, partially described in 3, not in 6. Allocation concealment was carried out and reported in only 1 study, and was unclear in the remainder. The researcher was clearly masked to the intervention in 6 studies, the outcome assessors in 6 studies and participants in 13 studies, while in most of the remaining cases masking was unclear. There were dropouts in 9 studies (none in 4 studies, unclear in another, see Table 1), while all those randomised were included in outcomes in one study (unclear in 2, not in the remainder, see Table 2), 3 studies fully reported the reasons for dropouts and 5 studies partially reported them. There was moderate potential for funding bias in 9 studies, a high risk of funding bias in 4 studies and a low risk of funding bias in only one study (see below Table 2 for details of how this was assessed). Iron status changed in the intervention relative to the control group, or intervention lasted at least 12 weeks, in 20 of the 23 included arms, unclear in one, and not in two arms. Only one arm for which data were included in the meta-analysis was unclear about iron status change and so data from this study were excluded in sensitivity analyses [20].

### Outcomes

#### Attention/Concentration

**Figure 2:**
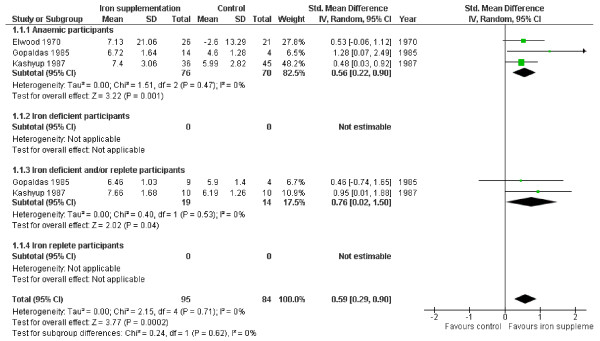
**Forest plot, SMD analysis of the effect of iron supplementation on attention and concentration**.

The effect of iron supplementation on attention or concentration was assessed in 3 groups of anaemic participants (146 people) and two groups of iron deficient and/or replete participants (33 people), see Figure 2. Iron supplementation appeared to have statistically significant beneficial effects on attention or concentration overall (SMD 0.59, 95% CI 0.29 to 0.90, p_effect _= 0.0002, n = 179, I^2 ^0%, p_heterogeneity _0.71) as well as in both subgroups, without any suggestion of heterogeneity. This standardized mean difference could be interpreted as a 'moderate effect' [28]. Improvement in attention and concentration occurs in the set of studies of children aged 6-18 (with no suggestion of heterogeneity), but the only study of pre-menopausal women does not suggest a statistically significant effect on its own (see Table 3). The effect is weakened, but remains statistically significant when the data from the one study that did not clearly improve iron status [20] were removed.

**Table 3:** Meta-analysis, subgrouping by age group. SMD analysis of the effect of iron supplementation on cognitive domains

Cognitive domain	Age group	Standardised mean difference (95% CI)	Number of participants/studies	Heterogeneity - I^2 ^test
Attention & concentration	Children aged 6-18	0.62 (0.26 to 0.98)*	132/2 (4 arms)**	0%
	
	Pre-menopausal women	0.53 (-0.06 to 1.12)	47/1	Not relevant

Intelligence	Children aged 6-18	0.02 (-0.22 to 0.27)	2289/4 (9 arms)**	82%
	
	Pre-menopausal women	0.62 (0.15 to 1.10)*	76/2	0%

Memory	Children aged 6-18	0.33 (-0.19 to 0.85)	132/2 (4 arms)**	35%
	
	Pre-menopausal women	0.09 (-0.31 to 0.50)	123/3	18%

Psychomotor	Children aged 6-18	0.19 (-0.17 to 0.54)	132/2 (4 arms)**	0%
	
	Pre-menopausal women	0.09 (-0.32 to 0.50)	123/3	21%

Scholastic aptitude	Children aged 6-18	0.03 (-0.63 to 0.69)	1799/3 (6 arms)**	96%
	
	Pre-menopausal women	0.77 (-0.08 to 1.62)	25/1	Not relevant

* p < 0.05

** Two studies included 3 sets of participants defined by baseline iron status [15,24], and two further studies included 2 sets of participants defined by baseline iron status [21,23], these arms were entered separately into the meta-analysis.

We located five studies that fulfilled the review inclusion criteria, assessed effects of iron on attention and concentration, but which provided data in a format that could not be included in meta-analysis (5 studies, including 8 intervention arms). One study in Indonesian primary school children [29] found an improvement in attention and concentration related to iron supplementation, while the remaining four studies (in US adolescents and pre-menopausal women, New Zealand teenagers and Mexican primary school children) found no statistically significant effects on measures of attention or concentration [27,30-32]. It is not clear whether inclusion of the data from these five studies would have reduced or reinforced the suggested improvement in attention and concentration with iron supplementation.

#### Intelligence

**Figure 3:**
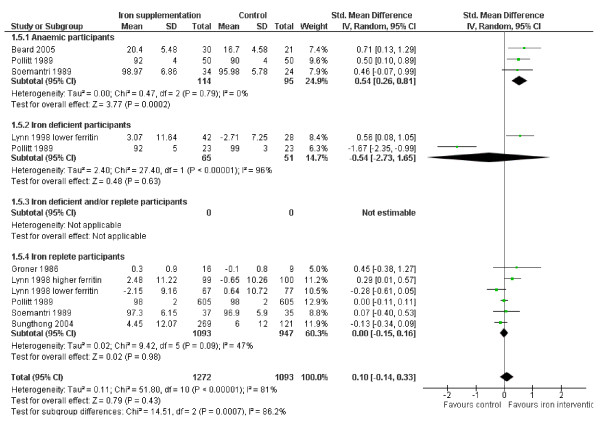
**Forest plot, SMD analysis of the effect of iron supplementation on intelligence**.

The effect of iron on intelligence quotient (IQ) was assessed in three groups of anaemic participants, two of iron deficient people and six groups of those who were iron replete at baseline, see Figure 3. Overall there was no evidence of an effect of iron supplementation on intelligence (SMD 0.10, 95% CI -0.14 to 0.33, p_effect _= 0.43, n = 2365, I^2 ^81%, p_heterogeneity _<0.00001), but there was evidence of an effect on people who were anaemic at baseline (SMD 0.54, 95% CI 0.26 to 0.81, p_effect _= 0.0002, n = 209, I^2 ^0%, p_heterogeneity _0.79), a moderate effect. These anaemic participants included African mothers with young children and school aged children from Thailand and Indonesia. Subgrouping by age group does not suggest any effect on intelligence in children, but does suggest a positive effect of iron supplementation in pre-menopausal women, with no heterogeneity (Table 3). All three studies used Raven's Colour Progressive Matrices to measure IQ. We transformed the unitless SMD back to the IQ scale used with Raven's Colour Progressive Matrices by multiplying it by the among-person standard deviation for the scale within the Pollitt 1989 study (as this was the largest study in anaemic participants), and based the variance on the pooled standard deviation of the same study as suggested by the Cochrane Handbook, section 12.6.4 [16]. The effect of iron supplementation in those with anaemia was equivalent to an improvement of 2.50 (MD 2.50, 95% CI 1.24 to 3.76) in IQ as compared with placebo.

The data were highly heterogeneous in the few iron deficient participants and suggested no effect in the more than 2000 participants who were iron replete at baseline. Sensitivity analysis, removing the study where it was not clear whether iron status improved with supplementation, did not alter the overall non-significance of the effect of iron supplementation on intelligence, or any of the subgroups.

**Figure 4:**
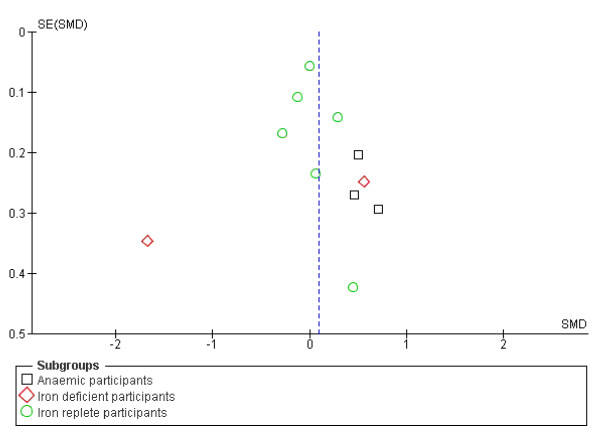
**Funnel plot, based on studies with data on intelligence**.

To assess the likelihood of publication bias being present in the data we created a funnel plot using the data on intelligence (as this was the outcome with most studies included in meta-analysis). The funnel plot did suggest some risk of publication bias, see Figure 4, but it is not clear whether this could have been accounted for by the studies that we located but that could not be included in the meta-analysis. The three included studies (six intervention arms, of which two were in anaemic participants) that assessed the effect of iron supplementation on intelligence but could not be included in the analysis all found no statistically significant differences in intelligence between the intervention and control groups. It is unclear how inclusion of these data would affect the overall results.

#### Memory

**Figure 5:**
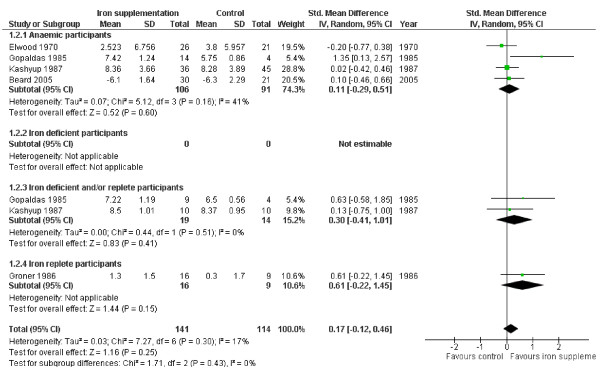
**Forest plot, SMD analysis of the effect of iron supplementation on memory**.

Four studies addressed the effect of iron supplementation on memory in anaemic participants, none in iron deficient people, 2 in those iron deficient and/or replete and 1 in those iron replete at baseline, see Figure 5. There was no evidence overall (SMD 0.17, 95% CI -0.12 to 0.46, p_effect _= 0.25, n = 255, I^2 ^17%, p_heterogeneity _0.30) or in any subgroup, that iron supplementation improved memory. There was no suggestion of important heterogeneity, and removing the one study [20] without a clear effect on iron status made no difference to the overall effect of iron. Subgrouping by age group suggested no differential effects by age (Table 3). Four further studies which measured effects on memory did not provide data for meta-analysis. One of these studies reported finding a statistically significant effect of iron supplementation on memory, the others did not suggest a statistically significant effect [30].

#### Psychomotor function

**Figure 6:**
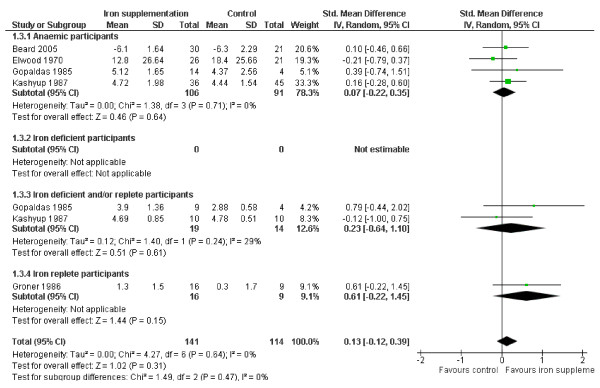
**Forest plot, SMD analysis of the effect of iron supplementation on psychomotor skills**.

The domain of psychomotor function was investigated by 4 studies in those anaemic at baseline, 2 in iron deficient and/or replete participants and 1 in iron replete participants (none in iron deficient participants), Figure 6. There was no suggestion of an effect of iron supplementation on psychomotor function overall (SMD 0.13, 95% CI -0.12 to 0.39, p_effect _= 0.31, n = 255, I^2 ^0%, p_heterogeneity _0.64) with no suggestion of heterogeneity. Similarly there was no significant effect on the subgroups of anaemic or iron replete participants, or in subgroups of children or pre-menopausal women. Removing studies without a clear effect on iron status did not alter these results. One study which did not provide data for the meta-analysis measured psychomotor function, and found no effects on it of iron supplementation [30].

#### Scholastic achievement

**Figure 7:**
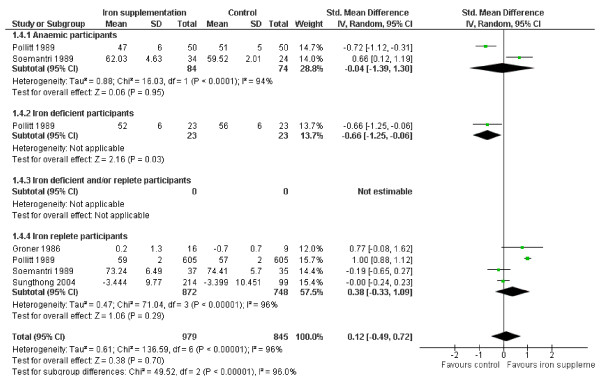
**Forest plot, SMD analysis of the effect of iron supplementation on scholastic ability**.

The effects of iron supplementation on scholastic achievement appeared highly heterogeneous - two studies showed statistically significant improvement, and two showed statistically significant impairment to scholastic achievement from iron supplementation compared with the control group at end of study. Overall, there was no suggestion of a significant effect (SMD 0.12, 95% CI -0.49 to 0.72, p_effect _= 0.70, n = 1824, I^2 ^96%, p_heterogeneity _<0.00001), Figure 7. There was no significant effect in the subgroup of participants with anaemia at baseline, or who were iron replete at baseline, or in children or pre-menopausal women, but in the one study in iron deficient participants iron appeared to worsen scholastic achievement. Removing studies where the effect of supplementation on iron status was not clear resulted in no changes in statistical significance except that the one study remaining in the subgroup with anaemia at baseline suggested a harmful effect of iron supplementation on scholastic achievement. Three studies which did not provide data for the meta-analysis assessed effects on scholastic achievement. One study found significant improvements [29], the others found no effects of iron supplementation.

#### Adverse effects

Adverse effects of iron supplementation were not well reported in the included studies, with the exception of Bruner et al [30], which mentions 'constipation'. In the 1475 participants randomised within studies that reported dropouts by arm, there was a relative risk of dropping out of 0.80 (95% CI 0.62 to 1.03) in iron supplemented compared to placebo arms.

## Discussion

This systematic review of 14 studies has assessed the effects of iron supplementation on cognition in males and females aged 6 years and older. The participants of the included studies were most often children or adolescents (10 studies, of which 7 were from developing countries). The remaining studies were in women, generally younger women - only one study included women over 35 years old. No studies included men, post-menopausal women or the elderly.

We found some evidence that iron supplementation improved attention and concentration in adolescents and women at all levels of iron status at baseline over periods of 8-17 weeks. Iron supplementation also improved IQ in children and women with anaemia at baseline over 13-29 weeks, but had no effects on memory, psychomotor skills or scholastic achievement. However, most studies were small, methodologically weak and there was evidence of publication bias.

There were over 1500 children and adolescents in the iron replete subgroup assessing effects on both intelligence and scholastic ability, suggesting that this group was adequately powered to detect an effect, and that the lack of effect observed in these iron replete samples is likely to be reliable over 4-29 weeks. However, the included studies were of short duration and for all outcomes effects may be greater, or different, in the longer term. In other subgroups where no effects are seen, this may be due to a lack of power and/or short duration, making it less likely that any true effects can be discerned.

### Comparison with other literature

Benton found repeated and consistent reports in both developing and developed countries of associations between iron status and intellectual ability or scholastic performance, with more subtle effects with less severe iron deficiency [33]. A previous review found that iron supplementation appeared to improve mental development scores in older children, but did not address the different domains of cognition [8]. We have extended this analysis, confirming that iron supplementation appears to improve attention and concentration in older children and adults and improves certain measures of intelligence quotient in those with anaemia at baseline. However, there is no evidence that other cognitive domains are affected by iron supplementation.

Severe anaemia results in increased mortality in women and babies [2,4]. A large and comprehensive systematic review of the effect of routine oral iron supplementation during pregnancy included 40 RCTs or quasi-randomised trials, but did not identify cognitive outcomes in mothers [34]. No other systematic reviews of the effects of iron supplementation on cognition in adults were identified, although there is reasonable evidence of the effects of iron deficiency on work capacity, suggesting that IDA reduces aerobic capacity, with less clear effects on endurance capacity and voluntary activity [35].

As in previous reviews, no RCTs assessing the effect of iron supplementation on cognition in the elderly were found [36], and data on adults generally were scarce. A systematic review found only one case control study that addressed the relationship between anaemia and cognition [36], finding that Alzheimer's disease was twice as prevalent in older people with anaemia. Another more recent systematic review of three longitudinal studies found a doubling of the risk of dementia in those with anaemia [37]. This was confirmed by a recent study which suggested that IDA is associated with poorer cognitive function over and above the already elevated risk of cognitive decline in this group [38].

When data were combined from studies in children and pre-menopausal women the lack of heterogeneity between studies assessing attention and concentration suggested that similar mechanisms may be determining the effects of iron supplementation on cognition across these age groups.

### Strengths and weaknesses of the review

Study duration of included studies is of concern, the shortest included studies were only 4 weeks in duration, and five included studies were shorter than 12 weeks. Twelve weeks of supplementation is sufficient to alter iron status, and so alter oxygen supply to the tissues, but shorter studies may not be long enough to ensure this has occurred. It is possible that including studies of too short duration will dilute effects, and potentially negate any effect of iron on cognition. To check this we performed sensitivity analysis, removing studies that did not show statistically significant improvements in Hb or SF in the intervention group compared to the control, or were shorter than 12 weeks duration. This did not alter either the significant effects on the attention and concentration or intelligence, or the lack of significance in other groups, suggesting that the included studies were long enough to ensure improved iron status in intervention arms.

However, for outcomes such as scholastic achievement, improvement may require a much longer intervention period than the time necessary to replete Hb levels. Even when SF and/or Hb concentration has improved, a further period may be required for performance improvement to occur. This is particularly relevant in relation to scholastic achievement where iron status at learning may be different from iron status at retrieval of information or assessment of performance. This could result in a lack of effect of iron supplementation being detected on tests of this type. For these outcomes even studies of 29 weeks (the longest of our included studies) may not be sufficient to see important effects. The effects of longer term studies are unclear and this is an important area for future research.

Adverse effects have been associated with iron supplementation, for example increasing the risk of developing diarrhoea [10,39] or constipation. Failure to document the type or prevalence of adverse effects in the included studies of this review makes it harder to assess the acceptability of iron supplementation for the target groups, or to begin to address the balance of risk and benefit. However, the lack of an excessive risk of dropout in the participants taking iron supplements compared with placebo suggests that any experienced side effects were not severe enough for participants to cease participating, although they may have surreptitiously reduced their intake of the iron supplements. Compliance was not well addressed in the included studies.

A range of cognitive tests were used in the studies reviewed. The cognitive domain assessed by each test was determined on the basis of the description of the test features. These were not always sufficiently detailed to permit confident classification. Some researchers classified ostensibly similar tests as measuring quite different cognitive domains. For instance the 'E-test' carried out in Elwood [20] and the 'clerical task' carried out in the studies by Gopaldas and Kashyup [21,23] are, on the basis of their description, very similar. However, the E-test is reported to be a test of 'vigilance, concentration and a degree of dexterity', while the clerical task is said to test 'attention, concentration and discrimination'. Moreover, the tests used in the cognitive domains of attention and vigilance are similar in some aspects to Raven's Colour Progressive Matrices which, although a proxy for IQ and classified as such here, also showed positive effects of iron supplementation in those with anaemia. We addressed this by allocating the domains ourselves from the descriptions of the tests, independently of classifications provided in the published papers.

A large number of cognitive measures were employed across the studies with some cognitive domains examined more frequently than others e.g. tests of verbal memory and IQ and attention were most common. Not all studies assessed more than one aspect of cognitive function and the timing of tests post-intervention also varied. The cognitive tests employed in the studies were fairly limited, and these were not necessarily selected for their sensitivity to nutrient intervention or change over time. Some studies used global neuropsychological tests, more usually employed for diagnostic purposes or to ascertain a stable measure of intellectual function. Across studies, tests were not readily comparable and accuracy and error rates were not provided by all studies, and the validity, reproducibility or cultural/language appropriateness of these tests were rarely discussed. To partially address these issues we restricted the outcomes assessed in this review to the most objective and valid available in the literature (excluding for example teacher or parent ratings of behaviour, which can be highly volatile), but outcome measures were not ideal.

Although ecologically valid, end of year school performance may not provide the most sensitive indicator of the effect of iron supplementation and many studies which used scholastic performance as an outcome did not control for other factors which are likely to influence school grade, including home environment, parental involvement, school system and quality. The nature of the testing situation is important. Teacher or researcher administered tests, especially where the tester is not blind to the treatment arm, may positively influence the performance of the active treatment group [40]. Computerised, individual and blind testing can minimise these experimenter effects. With such limited numbers of included studies there were too few data to address the effects of specific types of test or types of administration.

A recent systematic review of the effects of breakfast on cognitive performance [41] concluded that breakfast consumption improved verbal fluency and memory tasks in nutritionally vulnerable children, particularly short term recognition, memory search and measures of visual perception. These verbal fluency and memory tasks, which appeared susceptible to nutritional intervention, were not well represented in the studies reported here. Moreover, little consideration was given to motivation and effort including the ability to sustain performance over time which might be influenced by long term supplementation or indeed study participation. Sustaining concentration and retaining information are cognitive processes of key importance for scholastic achievement or other long measures of performance. This may be a partial explanation of why positive effects of iron supplementation were clearest in those with deficiencies which were corrected by the intervention.

Five studies were identified that could not be included in the meta-analyses. This was because the outcomes were reported as z-scores or were adjusted (both of these ways of analysing the data are appropriate, but they render the data incomparable in meta-analysis), or because of a lack of variance data. Inclusion of the results of these studies in the meta-analyses, had we been able to retrieve these data in an appropriate format, could have either reinforced or negated the results of the analyses. This, along with some suggestion of publication bias (see Figure 4) suggests that the true effect of iron supplementation on cognitive outcomes is unclear.

In some studies (where SF had not been measured) it was not clear that anaemia was due to iron deficiency, however results did not alter when the one study which did not show an effect of iron supplementation was removed. Another area of uncertainty was the nutritional status of participants aside from iron status, which was assessed in most studies. Iron supplementation may be less effective where there are a number of nutritional problems at baseline (all of which may be contributing to cognitive limitations) than where participants are nutritionally replete except for variations in iron status. For example, iron and zinc deficiencies often occur together, and zinc deficiency can be exacerbated with high dose iron supplements [42]. As zinc may also play a role in cognitive function, iron supplementation could exacerbate cognitive deficits [43]. This may be reflected in different effects in developing compared to developed countries, but is more likely to reflect differences between individuals within the studies. A related issue, raised by the late Professor John Beard when he replied to our requests for further information on one of his included studies, was whether an intention to treat analysis of the data is valid, or whether we should be assessing the effects of iron supplementation only in individuals whose iron status demonstrably improves. This is a well-worn argument between analysis by intention to treat (effectiveness) and by per protocol analysis (efficacy), and the two types of analysis answer different questions. The intention to treat analysis, where all those randomised to the intervention are analysed (and compared to all those randomised into the control group) assesses the effectiveness of an intervention (in this case iron supplementation) on the whole group of potential recipients. It takes into account that some individuals may not take the treatment for a variety of reasons, and some may experience side effects, but assesses the effect overall in the whole group. The per protocol analysis would assess efficacy - the effect only in those individual participants who clearly respond to treatment with a Hb or SF rise (and would omit those who experience such increases in the control group), so is assessing the effect of a specific improvement in biomarkers of iron status as functional iron (Hb) or storage iron (SF), rather than the overall effectiveness of supplementation. The difficulty with this approach is that before providing the supplement it is not possible to predict whether any one individual will respond with the required iron status change. Several people may have to be supplemented to assess effects in just one person. Assuming that there is a relationship between iron status and a cognitive domain, the per protocol approach is more likely to identify the effect with small sample sizes, but will also overstate the effect size when a population are considered as a whole [44]. Not enough studies carried out a per protocol analysis for us to carry out an alternative analysis on this basis in the review, although it would have been interesting to do this. Overall, it is our view that, as individual response to iron treatment (efficacy) cannot be pre-judged, that an intention to treat analysis (effectiveness) is the more useful when considering treatment of an at-risk group, but a per protocol analysis of small studies may help in understanding whether a larger RCT of such a group using an intention to treat analysis would be worthwhile.

## Summary

We found some evidence that iron supplementation improved attention and concentration in adolescents and women, regardless of baseline level of iron status. Iron supplementation also improved IQ in women and children who were anaemic at baseline, but had no effect in other groups or on other cognitive domains. Further well powered, blinded and independently funded studies of at least one year's duration in children, adolescents, adults and older people with varying levels of baseline iron status and using well validated tests of cognition are needed to confirm and extend these results.

## Abbreviations

AC: Attention and concentration; EFe: elemental iron; IDA: iron deficiency anaemia; Hb: haemoglobin; IQ: intelligence quotient; M: memory; MD: mean difference; Ps: psychomotor; RCT: randomised controlled trial; SA: Scholastic Achievement; SF: serum ferritin; SMD: standardised mean difference; TS: transferrin saturation

## Ethical approval

As our research was a systematic review (secondary research, not involving any contact with people or patients directly, but instead a thorough detailed assessment and analysis of the data from a set of published primary research) ethical approval was not necessary.

## Competing interests

The authors declare that they have no competing interests.

## Authors' contributions

PC and LH devised the review; PC carried out initial searches and MF carried out updating searches; PC, LH, MF and AA checked lists of titles and abstracts resulting from electronic searches, assessed full text studies for inclusion, extracted data and assessed validity; LD provided expertise in cognitive assessment and SF-T in iron metabolism throughout; MF and PC developed the tables; MF wrote the first draft of the manuscript and LH developed the manuscript for publication; all authors were involved in critical discussion and editing the manuscript for publication, and all authors agreed the final text.

## Supplementary Material

Additional file 1 PRISMA 2009 Checklist Click here for file

Additional file 2 Database: Ovid MEDLINE Click here for file
