# Supplementary material for: The effects of oral iron supplementation on cognition in older children and adults: a systematic review and meta-analysis
Source: Nutr J. 2010 Jan 25;9:4. doi: 10.1186/1475-2891-9-4 (PMC2831810; doi:10.1186/1475-2891-9-4)
Supplement: Additional file 2 — Database: Ovid MEDLINE [file 1475-2891-9-4-S2.DOC]

Database: Ovid MEDLINE(R) <1950 to October Week 4 2008>

Search Strategy:

--------------------------------------------------------------------------------

1 randomized controlled trial.pt.

2 controlled clinical trial.pt.

3 randomized controlled trials/

4 random allocation/

5 double-blind method/

6 single-blind method/

7 6 or 4 or 1 or 3 or 2 or 5

8 animal/ not human/

9 7 not 8

10 exp Cognition/

11 learning/ or cues/ or exp memory/ or problem solving/ or exp verbal learning/

12 perception/ or space perception/ or exp visual perception/

13 exp Thinking/

14 exp Psychomotor Performance/

15 Neurodegenerative Diseases/

16 exp Dementia/

17 exp Dementia, Vascular/

18 exp Parkinsonian Disorders/

19 neurodegenerative.mp.

20 (cognit$ or memor$ or mmse or adas-cog).mp.

21 Alzheimer Disease/

22 11 or 21 or 17 or 12 or 20 or 15 or 14 or 18 or 19 or 10 or 13 or 16

23 exp Iron/ or exp Iron Chelating Agents/ or exp Anemia, Iron-Deficiency/ or exp Iron, Dietary/ or exp Iron Overload/ or exp Iron Metabolism Disorders/ or exp Iron-Binding Proteins/ or exp Iron Compounds/

24 Minerals/

25 (iron$ or fe or ferric or ferrous or fe2$ or anaemi$ or anemi$ or sprinkles).mp.

26 25 or 24 or 23

27 22 and 26 and 9
